# 静脉血栓栓塞疾病的遗传学研究进展

**DOI:** 10.3760/cma.j.cn121090-20240327-00117

**Published:** 2024-12

**Authors:** 云晴 夏, 亮 唐, 豫 胡

**Affiliations:** 华中科技大学同济医学院附属协和医院血液病研究所，武汉 430022 Institute of Hematology, Tongji Medical College Affiliated Union Hospital, Huazhong University of Science and Technology, Wuhan 430022, China

## Abstract

静脉血栓栓塞症（VTE）临床常表现为下肢深静脉血栓形成（DVT）和肺栓塞（PE）。VTE是仅次于冠状动脉和脑血管疾病的第三大常见血管疾病。VTE是一种多因素疾病，由遗传性、获得性危险因素相互作用引起。在对家庭、双胞胎、兄弟姐妹的研究中估计遗传率达40％～60％。随着基因测序技术的不断进步，将遗传变异与VTE风险联系起来的信息迅速积累，目前凝血系统、抗凝系统以及纤溶系统中关键基因的突变不断更新，除此之外，新基因的发现以及功能机制探究备受关注。本综述将总结概括VTE相关关键基因变异的研究进展及未来展望。

## Abstract (en)

Venous thromboembolism（VTE）is clinically manifested as deep vein thrombosis（DVT）and pulmonary embolism（PE）. VTE is the third most prevalent vascular disease after coronary artery and cerebrovascular diseases. VTE is a multifactorial disease caused by the interaction of genetic and acquired risk factors. Genetic heritability is estimated to be 40％–60％ based on studies of families, twins, and siblings. The accumulation of information linking genetic variations to VTE risk has rapidly expanded with the continuous advancement of sequencing technology. Currently, mutations in key genes of the coagulation system, anticoagulation system, and fibrinolysis system are constantly being updated, and the functional mechanisms of new genes are receiving gaining attention. This review summarizes the research progress and prospects of key genetic variations associated with venous thromboembolism.

静脉血栓栓塞症（VTE）临床常表现为下肢深静脉血栓形成（DVT）和肺栓塞（PE）。VTE是仅次于冠状动脉和脑血管疾病的第三大常见血管疾病[1]。VTE是一种多因素疾病，由遗传性、获得性危险因素相互作用引起。在对家庭、双胞胎、兄弟姐妹的研究中估计遗传率达40％～60％[2]。随着测序技术的不断进步，将遗传变异与VTE风险联系起来的信息迅速积累，目前凝血系统、抗凝系统以及纤溶系统中关键基因的突变不断更新，除此之外，新基因的发现以及功能机制探究备受关注。本综述将总结概括静脉血栓栓塞疾病相关关键基因变异的研究进展及未来展望。

一、凝血与抗凝机制

当血管内皮受到损伤时，机体凝血系统可通过血小板激活、黏附、聚集以及内源性、外源性凝血因子级联放大，最终形成局部高浓度的凝血酶，分解纤维蛋白原，形成血凝块止血，而抗凝系统分别作用于多种不同凝血因子使其灭活，限制凝血酶生成和血栓形成。正常机体的凝血、抗凝和纤溶系统处于动态平衡。各种凝血因子、抗凝因子和纤溶因子发生数量变化或功能障碍时，均可使凝血与抗凝平衡紊乱，从而导致出血或血栓形成。

二、凝血系统

（一）凝血因子Ⅴ（FⅤ）

FⅤ Leiden是VTE最常见的遗传危险因素，见于20％～25％的VTE患者和50％的家族性易栓症患者。其特征是对活化蛋白C（APC）的抗凝反应降低。FⅤ是指FⅤ基因中核苷酸1 691处特异性鸟嘌呤（G）被腺嘌呤（A）取代，即谷氨酰胺的APC切割位点被精氨酸取代。这种突变导致活化FⅤ（FⅤa）对APC具有抗性，并且失活的速度是正常速度的1/10，导致凝血酶生成增加。因此产生轻度血栓前反应为D-二聚体、凝血酶原片段F1+2和其他活化凝血标志物水平升高。最近的一项荟萃分析也证实了杂合FⅤ突变患者的VTE复发风险增加了46％[3]。近年来的研究还发现了FⅤ Nara（Trp1920Arg）、FⅤ Bonn（Ala512Val）等罕见的FⅤ基因突变，这些突变影响了FⅤa的功能，导致了携带突变的患者对APC耐药以及血栓形成[4]。

（二）纤维蛋白原

1. FGB基因：FGB基因突变值得关注，因为Bβ链被认为是纤维蛋白原六聚体肝脏生成的限速因子。因此，Bβ链的突变会影响正常的纤维蛋白原六聚体的分泌，导致纤维蛋白原定量失衡[5]。在携带FGB基因突变的患者中，即使低水平的突变纤维蛋白原也可能通过影响纤维蛋白凝块特性（如纤维蛋白溶解）而导致高凝状态[6]。目前报道的FGB突变有杂合错义Y416C、A68S，纯合无义Y345，以及杂合无义W403突变等，在扫描电子显微镜下也证实了这些突变会导致纤维密度的改变，影响正常纤维蛋白原的结构与功能[7]。

2. FGG基因：有数据统计纤维蛋白原突变80％位于FGG基因γ链[5]。目前报道的FGG基因7号外显子c.677G>T（p.Gly226Val）杂合错义突变导致p.Gly266残基暴露。由于p.Gly266残基与p.Tyr374侧链相互结合，破坏了该分子三级结构的稳定性。携带此突变的男童7个月大时因颅内血栓形成而死亡[8]。另外一项报道也是发生在FGG基因7号外显子c.823G>T（p.Glu275Stop）的杂合无义突变，γ链的C端结构域对肝细胞分泌纤维蛋白原具有至关重要的作用，这种无义突变极有可能会导致纤维蛋白原分子不能进行分泌，同时，也不排除一些转录本跳过终止密码子的替代机制，从而分泌具有血栓形成潜力的纤维蛋白原[9]。Paulsen等[10]最近的一项队列研究证明了携带变异FGG纯合子癌症患者发生VTE的风险是无FGG变异的癌症患者的两倍，在变异FGG纯合子携带者中，癌症患者VTE的6个月累积发病率为6.4％（95％*CI* 3.5％～11.6％），而没有风险等位基因变异的患者中VTE的6个月累积发病率为3.1％（95％*CI* 2.3％～4.7％），这提示了FGG基因突变在癌症相关血栓疾病中具有重要意义。

（三）凝血酶原

凝血酶原G20210A（c.97G>A）突变是静脉血栓形成的常见危险因素，主要通过因子Ⅱ（FⅡ）血浆水平升高介导。研究统计，FⅡ G2021A变异可导致静脉血栓形成风险增加近3倍[11]。2012年首次报道了引起抗凝血酶抵抗效应的凝血酶异常。随后在塞尔维亚、印度和意大利等国家相继发表了类似的家族聚集性病例[12]。这些家族涉及同一个氨基酸上的三个不同突变：凝血酶原Arg596Leu（Yukuhashi）、Arg596Gln（Belgrade）和Arg596Trp（Padua）。值得注意的是，所有这些患者都是突变的杂合子，间接证实了凝血酶原缺陷的同基因遗传非常严重，这些异常病例与血栓倾向有关。最近的一项研究中，新的杂合凝血酶原突变Arg541Trp通过损害蛋白C通路功能以及增强凝血酶的促凝活性而成为VTE新的遗传危险因素[13]。

三、抗凝系统

（一）PROC和PROCR基因

遗传性蛋白C缺乏症是由位于染色体2q14.3上的蛋白C基因（PROC）突变引起，其中错义和无义突变最常见。严重的蛋白C缺乏症（纯合子或复合杂合子形式）极其罕见（人群发病频率为1/750 000～1/500 000），但部分缺乏症（杂合子形式）是常见（1/200～1/500）突变类型[14]。杂合蛋白C缺乏症可分为Ⅰ型（蛋白C抗原水平和活性均降低，占75％～80％）和Ⅱ型（蛋白C抗原水平正常，活性降低）[14]。Tang等[15]在34例遗传性蛋白C缺乏症先证者中发现了18种突变，其中常见突变c.565C>T导致一级亲属患静脉血栓形成的风险增加了8.8倍。此外，在另一项研究中对32例蛋白C缺乏症患者进行测序分析，发现10例患者携带PROC c.574_576del变异[16]。在丹麦人群中的PROC基因变异研究中，从13个先证者的PROC基因检测到四种（c.503T>C、c.710C>A、c.1000G>A和c.1015G>A）可能致病性变异[17]。Lu等[18]发现c.715G>A（p.Gly197Arg）变异被凝血酶激活，并且血栓调节蛋白（TM）在促进其活化方面没有辅因子活性。此外，APC此突变体的抗炎活性也因内皮细胞的通透性改变而显著受损。近期一项研究发现PROC基因第7外显子c.572_574delAGA（p.Glu191_Lys192delinsGlu）杂合缺失和第8外显子c.752C>T（p.Ala251Val）杂合错义变异可能是遗传性蛋白C缺乏的病因之一[19]。迄今为止，全球已报告了400多种PROC基因突变，大多数是单碱基置换，少数为缺失或插入[20]。一项基于人群的队列研究在28 794名没有先前VTE受试者中确定了PROCR基因Ser219Gly变体和Arg113Cys错义变体显著增加了VTE风险[21]。

（二）PROS基因

遗传性蛋白S（PS）缺乏症是一种具有常染色体显性遗传模式的遗传性血栓疾病。全球已报道了453个定位在蛋白S基因（PROS1）的变异。其中，错义/无义突变占61％。Banno等[22]研究发现PROS1基因c.586A>G（p.Lys196Glu）突变加剧了小鼠静脉血栓栓塞的发生。Fujita等[23]在一例54岁突发全身性癫痫的脑静脉血栓患者中发现了PS-K196E杂合突变，也证实了此突变在VTE中的风险。Zhang等[24]在肺栓塞患者中检测到一种新的PROS1移码突变c.74dupA，并且他的母亲也携带此突变并患有蛋白S缺乏症。Wang等[25]发现了PROS1 IVS10+5G>A突变，改变了PROS1基因的正常剪接过程，可能是一种致病突变。

（三）SERPINC1基因

抗凝血酶Ⅲ（AT-Ⅲ）是抗凝系统中的重要成分，由肝脏合成，为一种丝氨酸蛋白酶抑制物，可抑制凝血酶生成。编码AT-Ⅲ的基因SERPINC1突变导致抗凝血酶缺乏症可分为Ⅰ型（定量缺陷）和Ⅱ型（定性缺陷）。1983年第一个抗凝血酶缺乏变异的定性标志着血栓形成风险遗传基础研究的开始。多年来，已有200多个与血栓风险相关的变异被报道。一个值得注意的变异是SERPINC1基因中的Phe229Leu突变，它与体内自发性抗凝血酶聚合有关，并与严重的儿童血栓形成相关。并且，该变异体的杂合型主要与静脉血栓风险增高有关[26]。在已发现的变异中，SERPINC1基因中rs2227589多态性备受关注。在一项荷兰人群中进行的研究发现，该多态性与静脉血栓风险相关，其等位基因频率在gnomAD数据库中为0.1，在东亚人群中为0.329[27]。rs2227589对抗凝血酶水平的功能影响已在正常西班牙白种人得到证实，这表明它在血栓形成易感性方面的潜在相关性。最新的研究发现了SERPINC1的新的无义突变c.637C>T（p.Gln213Ter）、错义突变c.1A>G（p.Met1Val）、c.1277C>T（p.Ser426Leu）等[28]–[29]。这些突变都与严重的易栓症相关，新突变的发现对VTE的风险筛查提供了新的依据。

四、纤溶系统

编码组织型纤溶酶原激活剂（t-PA）的基因PLAT-7351C>T单核苷酸多态性（rs63020761）位于增强子区域内，在-7351位胸腺嘧啶取代胞嘧啶会降低转录因子Sp1/Sp3的结合亲和力，导致DNA转录受到抑制。最近一项研究发现PLAT的罕见纯合点突变c.1411T>C（p.Y471H），t-PA的氨基酸471位从酪氨酸突变为组氨酸。此突变影响了PAI-1的结合点，导致氢键相互作用降低，自由结合能力降低，亲和力减弱，从而加剧血栓形成[30]。编码尿激酶型纤溶酶原激活剂（u-PA）的基因PLAU的1 788位的胞嘧啶转变为胸腺嘧啶（rs2227564），导致u-PA蛋白的Kringle结构域发生错义突变（Pro141Leu），已被证实会降低对纤维蛋白的亲和力，从而增加血栓栓塞的风险[31]。

五、新易感基因

以往的研究都是针对血栓相关已知基因的突变点展开，因而一些未知基因对VTE的影响可能被疏忽，这些位点可能不属于凝血或者纤溶通路，但在血栓形成中起到一定的作用。在过去的几十年中，发表了一系列全基因关联研究（GWAS），连续研究了更大的样本量（更大的人群）和更多的变异。尽管早期GWAS很重要，但无法鉴定与VTE相关的新候选基因。2015年，国际静脉血栓形成网络联盟（INVENT）研究人员对关于VTE的GWAS研究中的6 751 884个变异进行荟萃分析，其中包括7 507例患者和52 632名对照，除了发现6个以前已知的基因位点（ABO、F2、F5、F11、FGG和PROCR）外，还发现了3个新的基因位点，其中ZEPM2、TSPAN15和SLC44A2的变异具有统计学意义[32]–[33]。2019年在《Blood》发表的基因组和转录组关联研究进一步增加了与VTE相关的新基因位点的数量，这些新关联的遗传位点包括C1orf198、PLEK、OSMR-AS1、NUGGC/SCARA5、GRK5、MPHOSPH9、ARID4A、PLCG2、SMG6、EIF5A和STX10等。同年《Nature Genetics》杂志也发表了一项在百万退役军人和英国生物库中进行的一项发现性全基因组关联研究，测试了约1 300万个DNA序列变异与VTE的关联性（26 066个病例，624 053名对照），发现了22个新的基因位点[34]。最近的一项研究对393例无诱因VTE患者和6 114名对照者进行了全外显子组测序，发现STAB2存在罕见致病性变异，确定了STAB2存在新的VTE关联，印证了先前GWAS描述的与血管性血友病因子和因子Ⅷ的血浆水平相关[35]。

六、展望

VTE具有重要的遗传成分。在过去的几十年中，随着许多导致高凝状态的基因变异的鉴定，该领域的研究不断发展。目前，尽管遗传性易栓症的检测只能以高度选择性的方式进行，但更深入地了解遗传性风险因素仍然很重要，能够更好地预测风险，以便为具有遗传变异风险的患者做出循证决策。遗传和生物信息学技术的进步可能有助于在易栓症家庭和基于人群的病例对照研究中发现更多的遗传性易栓缺陷。

## References

[1] Duffett L (2022). Deep Venous Thrombosis[J]. Ann Intern Med.

[2] Heit JA, Phelps MA, Ward SA (2004). Familial segregation of venous thromboembolism[J]. J Thromb Haemost.

[3] Eppenberger D, Nilius H, Anagnostelis B (2022). Current knowledge on factor V leiden mutation as a risk factor for recurrent venous thromboembolism: a systematic review and meta-analysis[J]. Front Cardiovasc Med.

[4] Shimonishi N, Ogiwara K, Yoshida J (2023). Impaired factor V-related anticoagulant mechanisms and deep vein thrombosis associated with A2086D and W1920R mutations[J]. Blood Adv.

[5] Simurda T, Brunclikova M, Asselta R (2020). Genetic variants in the FGB and FGG genes mapping in the beta and gamma nodules of the fibrinogen molecule in congenital quantitative fibrinogen disorders associated with a thrombotic phenotype[J]. Int J Mol Sci.

[6] Asselta R, Robusto M, Platé M (2015). Molecular characterization of 7 patients affected by dys- or hypo-dysfibrinogenemia: Identification of a novel mutation in the fibrinogen Bbeta chain causing a gain of glycosylation[J]. Thromb Res.

[7] Ceznerová E, Kaufmanová J, Sovová Ž (2022). Structural and functional characterization of four novel fibrinogen mutations in FGB causing congenital fibrinogen disorder[J]. Int J Mol Sci.

[8] Davis RL, Mosesson MW, Kerlin BA (2007). Fibrinogen Columbus: a novel gamma Gly200Val mutation causing hypofibrinogenemia in a family with associated thrombophilia[J]. Haematologica.

[9] Simurda T, Caccia S, Asselta R (2020). Congenital hypofibrinogenemia associated with a novel heterozygous nonsense mutation in the globular C-terminal domain of the γ-chain (p.Glu275Stop)[J]. J Thromb Thrombolysis.

[10] Paulsen B, Skille H, Smith EN (2020). Fibrinogen gamma gene rs2066865 and risk of cancer-related venous thromboembolism[J]. Haematologica.

[11] Ghouse J, Tragante V, Ahlberg G (2023). Genome-wide meta-analysis identifies 93 risk loci and enables risk prediction equivalent to monogenic forms of venous thromboembolism[J]. Nat Genet.

[12] Miyawaki Y, Suzuki A, Fujita J (2012). Thrombosis from a prothrombin mutation conveying antithrombin resistance[J]. N Engl J Med.

[13] Wu X, Dai J, Xu X (2020). Prothrombin Arg541Trp mutation leads to defective PC (Protein C) pathway activation and constitutes a novel genetic risk factor for venous thrombosis[J]. Arterioscler Thromb Vasc Biol.

[14] Dinarvand P, Moser KA (2019). Moser protein C deficiency[J]. Arch Pathol Lab Med.

[15] Tang L, Guo T, Yang R (2012). Genetic background analysis of protein C deficiency demonstrates a recurrent mutation associated with venous thrombosis in Chinese population[J]. PLoS One.

[16] Tang L, Lu X, Yu JM (2012). PROC c.574_576del polymorphism: a common genetic risk factor for venous thrombosis in the Chinese population[J]. J Thromb Haemost.

[17] Winther-Larsen A, Kjaergaard AD, Larsen OH (2020). Protein C deficiency; PROC gene variants in a Danish population[J]. Thromb Res.

[18] Lu Y, Giri H, Villoutreix BO (2020). Gly197Arg mutation in protein C causes recurrent thrombosis in a heterozygous carrier[J]. J Thromb Haemost.

[19] 陈 源, 石 佳旻, 黄 小夏 (2022). 一个PROC基因变异所致的遗传性蛋白C缺陷症家系的分析[J]. 中华医学遗传学杂志.

[20] Togashi T, Meguro-Horike M, Nagaya S (2020). Molecular genetic analysis of inherited protein C deficiency caused by the novel large deletion across two exons of PROC[J]. Thromb Res.

[21] Manderstedt E, Halldén C, Lind-Halldén C (2022). Thrombotic risk determined by protein C receptor (PROCR) variants among middle-aged and older adults: a population-based cohort study[J]. Thromb Haemost.

[22] Banno F, Kita T, Fernández JA (2015). Exacerbated venous thromboembolism in mice carrying a protein S K196E mutation[J]. Blood.

[23] Fujita K, Sonoda K, Miyata T (2019). Early Identification of Protein S K196E Mutation in a Patient With Cerebral Venous Thrombosis: A Case Report[J]. J Stroke Cerebrovasc Dis.

[24] Zhang Y, Yang H, Chen Q (2016). A novel PROS1 mutation, c.74dupA, was identified in a protein S deficiency family[J]. Thromb Res.

[25] Wang X, Tang N, Wang X (2019). PROS1 IVS10+5G>A mutation causes hereditary protein S deficiency in a Chinese patient with pulmonary embolism and venous thromboembolism[J]. Thromb Res.

[26] Navarro-Fernández J, de la Morena-Barrio ME, Padilla J (2016). Antithrombin Dublin (p.Val30Glu): a relatively common variant with moderate thrombosis risk of causing transient antithrombin deficiency[J]. Thromb Haemost.

[27] Bezemer ID, Bare LA, Doggen CJ (2008). Gene variants associated with deep vein thrombosis[J]. JAMA.

[28] Yu H, Gai X, Wang J (2022). Missense mutation of SERPINC1 (p.Ser426Leu) in a young patient presenting as refractory and recurrent venous thromboembolism: A case report[J]. Front Cardiovasc Med.

[29] Zeng M, Jia K, Liu M (2023). A novel mutation p.Met1Val in SERPINC1 gene causes hereditary antithrombin deficiency in a Chinese family with thrombotic disease[J]. Thromb Res.

[30] Tao Y, Ma J, Feng Y (2023). Tissue-type plasminogen activator (tPA) homozygous Tyr471His mutation associates with thromboembolic disease[J]. MedComm (2020).

[31] Oszajca K, Wroński K, Janiszewska G (2014). The study of t-PA, u-PA and PAI-1 genes polymorphisms in patients with abdominal aortic aneurysm[J]. Mol Biol Rep.

[32] Lindström S, Wang L, Smith EN (2019). Genomic and transcriptomic association studies identify 16 novel susceptibility loci for venous thromboembolism[J]. Blood.

[33] Thibord F, Klarin D, Brody JA (2022). Cross-Ancestry investigation of venous thromboembolism genomic predictors[J]. Circulation.

[34] Klarin D, Busenkell E, Judy R (2019). Genome-wide association analysis of venous thromboembolism identifies new risk loci and genetic overlap with arterial vascular disease[J]. Nat Genet.

[35] Desch KC, Ozel AB, Halvorsen M (2020). Whole-exome sequencing identifies rare variants in STAB2 associated with venous thromboembolic disease[J]. Blood.

